# Interrelations Between Ethical Leadership, Green Psychological Climate, and Organizational Environmental Citizenship Behavior: A Moderated Mediation Model

**DOI:** 10.3389/fpsyg.2019.01977

**Published:** 2019-08-28

**Authors:** Muhammad Aamir Shafique Khan, Du Jianguo, Moazzam Ali, Sharjeel Saleem, Muhammad Usman

**Affiliations:** ^1^School of Management, Jiangsu University, Zhenjiangw, China; ^2^Department of Management Sciences, University of Okara, Okara, Pakistan; ^3^Lyallpur Business School, Government College University, Faisalabad, Faisalabad, Pakistan; ^4^Department of Management Sciences, COMSATS University Islamabad, Lahore, Pakistan

**Keywords:** supervisors’ ethical leadership, green psychological climate, organizational environmental citizenship behavior, gender, China

## Abstract

Synthesizing theories of ethical leadership, psychological climate, pro-environmental behavior, and gender, first, we proposed and tested a model linking supervisors’ ethical leadership and organizational environmental citizenship behavior *via* a green psychological climate. Then, we tested the moderating effect of gender on the indirect (*via* a green psychological environment) relationship between supervisors’ ethical leadership and organizational environmental citizenship behavior. Time-lagged (three waves, 2 months apart) survey data were collected from 447 employees in various manufacturing and service sector firms operating in China. Data were analyzed using structural equation modeling, bootstrapping, and multigroup techniques to test the hypothesized relationships. The results showed a positive relationship between employee ratings of supervisors’ ethical leadership and organizational environmental citizenship behavior. Moreover, a green psychological climate mediates the relationship between supervisors’ ethical leadership and organizational environmental citizenship behavior. Importantly, the multigroup analysis revealed that gender moderates the indirect relationship (*via* green psychological climate) between supervisors’ ethical leadership and organizational environmental citizenship behavior. The study carries useful practical implications for policymakers and managers concerned about environmental sustainability.

## Introduction

Environmental degradation has become a serious threat for the inhabitants of our world, and business organizations are considered as one of the major contributors toward this threat (Stern, 2011; Swim et al., 2011, 2019; Aguinis and Glavas, 2012; Inoue and Alfaro-Barrantes, 2015; Peng and Lee, 2019). Therefore, prior research asserts the need for integrating social and environmental issues in business strategy and operations (Aguinis and Glavas, 2012; Bansal and Hoffman, 2012; Testa et al., 2016, 2018). Past research has made invaluable contributions by foregrounding the role that formal mechanisms, such as environmental safety and health management systems, monitoring activities, and operational control, play in accentuating social responsibility and environmental sustainability (Darnall and Edwards, 2006; Darnall et al., 2008; Heras-Saizarbitoria and Boiral, 2013; Leung and Rosenthal, 2019; Peng and Lee, 2019).

Despite these significant contributions, critical omissions in the literature need to be addressed to advance this line of research theoretically and empirically. First, several scholars have contended that formal control mechanisms are deficient in handling the environmental issues, which are intricate and unethical in nature (Barnett et al., 2005; Boiral et al., 2015; Saeed et al., 2018; Testa et al., 2018; Wang et al., 2018; Tuan, 2019; Woosnam et al., 2019). Instead, environmental issues can be addressed through employees’ discretionary ethical and altruistic efforts (e.g., Robertson and Barling, 2013, 2017; Afsar et al., 2016; Saeed et al., 2018; Testa et al., 2018). However, as noted by Robertson and Barling (2017), research on how managers can encourage employees to demonstrate pro-environmental behaviors is still in its infancy. Therefore, there have been growing calls for identifying leadership behaviors that encourage employees’ pro-environmental behaviors and reduce business organizations’ detrimental effects on the environment, without sacrificing profit (Aguilera et al., 2007; Boiral et al., 2015; Afsar et al., 2016; Saeed et al., 2018; Testa et al., 2018; Suganthi, 2019). To contribute to this nascent, yet growing field of employees’ pro-environmental behaviors, we mainly build on social learning theory (Bandura, 1977, 1986) to propose that ethical leadership – “the demonstration of normatively appropriate conduct through personal actions and interpersonal relationships, and the promotion of such conduct to followers” (Brown et al., 2005, p. 120) – positively shapes employees’ pro-environmental behaviors. Specifically, we predict that supervisors’ ethical leadership positively affects organizational environmental citizenship behaviors (OCBEs).

OCBE is defined as an “individual behavior that is discretionary, not directly or explicitly recognized by the formal reward system, and that in the aggregate, immediately benefits the natural environment, and indirectly through this means, contributes to the organization and benefits specific individuals” (Robertson and Barling, 2017, p. 58). OCBE includes different pro-environmental behaviors, including waste reduction at work, recycling, conserving energy, and encouraging coworkers to promote pro-environmental behaviors at the workplace (Robertson and Barling, 2017). We focused on OCBE, as past research, albeit limited to a few studies, shows that OCBE not only positively contributes to firms’ environmental performance but also improves firms’ financial performance (Swim et al., 2011; Magnus et al., 2012; Kennedy et al., 2015). The key rationale to considering ethical leadership is its central focus on ethics, and its features, such as altruism and social responsiveness that we argue can positively influence OCBE. Extant research has mainly studied ethical leadership in relationship with employees’ (un)ethical behaviors (e.g., Eisenbeiß and Brodbeck, 2014; DeConinck, 2015; Usman and Hameed, 2017; Men et al., 2018; Usman et al., 2018; Moore et al., 2019), but the value of ethical leadership as a predictor of employees’ pro-environmental behaviors, such as OCBE, have been glossed over. As environmental sustainability is a moral value and its pursuit requires ethical behaviors (Barnett et al., 2005), ethical leadership’s central focus on ethics (Brown et al., 2005) can have constructive influences on environmental sustainability. Moreover, OCBE entails employees’ discretionary initiatives and behaviors that are often not tied to formal structures and rewards (Paillé et al., 2014; Robertson and Barling, 2017). Therefore, our focus on the relationship between supervisors’ ethical leadership and OCBE is timely and relevant. Thus, this study advances the literature on ethical leadership by signifying the consequential potential of ethical leadership for enhancing OCBE and advancing OCBE literature by establishing ethical leadership as an important antecedent of OCBE.

Second, given the untapped nature of this research inquiry, we know little about the mediating mechanisms and the boundary conditions of the relationship between ethical leadership and OCBE, leaving it unknown why and when ethical leadership positively affects OCBE. To contribute to filling in these gaps, we suggest that a psychological green climate – “employees’ perceptions and interpretations of their organization’s policies, procedures, and practices regarding environmental sustainability” (Norton et al., 2012, p. 212) – mediates this link between supervisors’ ethical leadership and OCBE. The green psychological climate is considered because employees’ perception of green climate positively affects employees’ pro-environmental behaviors (Dumont et al., 2017; Norton et al., 2017; Zhou et al., 2018). On the contrary, employees’ perception that organizational policies and procedures do not support environmental sustainability discourages employees’ engagement in pro-environmental behaviors and deteriorates firms’ green performance (Seroka-Stolka and Lukomska-Szarek, 2016; Zientara and Zamojska, 2018; Tuan, 2019; Wang et al., 2019). We suggest that managers can encourage employees to demonstrate OCBE by instilling a sense among employees that organizational policies and practices are eco-friendly. By doing so, we signify the value of a psychological climate as a mechanism through which employees connect to and make sense of ethical leaders’ features, which promote different forms of ethical behavior, such as altruism, and a sense of responsibility toward society. Thus, examining the role of green psychological climate as a mechanism through which ethical leaders influence OCBE carries important implications for theory and practice.

To advance our knowledge of the boundary conditions of the relationship between ethical leadership and OCBE, we draw on social role theory (Eagly, 1987; Eagly and Wood, 2012) and socialization theory (Schahn and Holzer, 1990) to propose that gender moderates the indirect relationship (*via* psychological green climate) between supervisors’ ethical leadership and OCBE. Recent research emphasizes that gender should be taken into account while analyzing individuals’ pro-environmental behaviors, such as OCBE (Eisler et al., 2003; Xiao and McCright, 2015; Kennedy and Kmec, 2018; Vicente-Molina et al., 2018). Thus, incorporating gender into the framework linking ethical leadership, green psychological climate, and OCBE can enhance our understanding of the gender-based differential effects of social learning processes toward the environment.

## Theory and Hypotheses Development

### Leadership and Employees’ Pro-Environmental Behaviors

Past research has revealed several factors that positively contribute to employees’ pro-environmental behaviors. Pro-environmental work climates (Robertson and Carleton, 2018) and organizational justice for pro-environmental behaviors which enhance employees’ pro-environmental commitment (Tuan, 2019) are important predictors of employees’ pro-environmental behaviors. Green human resource practices (Saeed et al., 2018; Luu, 2019), incentive-based conservation (Ezzine-de-Blas et al., 2019), monetary rewards (Nelson and Quick, 2013; Young et al., 2015), training about various pro-environmental behaviors (Jones et al., 2012), managers’ feedback about employees’ pro-environmental performance (Carrico and Riemer, 2011; Young et al., 2015; Xu et al., 2017), and collective green crafting (Luu, 2019) are also important factors that positively affect employees’ pro-environmental behaviors.

Indeed, a plethora of studies have documented the important role of several leadership styles in shaping employees’ pro-environmental behaviors. According to Robertson and Barling (2013), if consistently and influentially applied, transactional leadership can positively influence employees’ pro-environmental behaviors. Transactional leaders assign responsibilities to their subordinates for achieving eco-friendly goals, monitor their pro-environmental performance, and importantly, reward them for their pro-environmental behaviors (Robertson and Barling, 2013). Spiritual leaders positively influence employees’ pro-environmental behaviors by encouraging self-directed moral values, enhancing their perception of meaningful work, and shaping a spiritual work environment (Afsar et al., 2016). Responsible leadership also inspires employees’ pro-environmental behaviors by giving them incentives and mobilizing them to achieve environmentally friendly goals (Zhao and Zhou, 2019). Green transformational leadership emphasizes green vision and shapes employees’ harmonious passion for pro-environmental behaviors that positively affects employees’ engagement in pro-environmental behaviors (Robertson and Barling, 2017; Wang et al., 2018). Environmentally specific charismatic leadership (Tuan, 2019) and environmentally specific servant leadership (Luu, 2019) have also been reported to have positive relationships with employees’ pro-environmental behaviors. Despite being insightful, previous studies have largely ignored the relationship between leadership and OCBE.

### Ethical Leadership and Organizational Environmental Citizenship Behavior

Ethical leadership has gained enormous attention in the mainstream management literature due to its focus on ethics (Brown et al., 2005; Resick et al., 2013; Men et al., 2018; Usman et al., 2018; Moore et al., 2019). Ethical leadership also positively influences several employees’ work-related attitudes, behaviors, and performance outcomes, such as ethical behavior, organizational commitment, work engagement, knowledge sharing, learning, psychological well-being, good citizenship, affective commitment, and job satisfaction (Chughtai et al., 2015; Ahn et al., 2018; Bavik et al., 2018; Byun et al., 2018; Usman et al., 2018). The key features of ethical leaders include: honesty, fairness, altruism, a two-way communication, ethical accountability, social responsiveness, and a sense of responsibility toward subordinates, customers, organizations, environment, and society (Brown et al., 2005; Chughtai et al., 2015; Moore et al., 2019).

A leader as a moral person and a moral manger are two key building blocks of ethical leadership (Brown et al., 2005; Byun et al., 2018; Moore et al., 2019). As a moral manager, an ethical leader exercises his/her power and authority to guard the interests of employees, the organization, and society at large by demonstrating ethically appropriate conduct and managing ethical accountability. That is, as the moral manager, the ethical leader ensures the implementation of ethical standards through punishment and reward system, holds employees accountable, and makes appropriate decisions to guard the interests and rights of various stakeholders, such as employees, the organization, and society (Brown et al., 2005; Byun et al., 2018; Zhang and Tu, 2018). As a moral person, the ethical leader demonstrates fairness, honesty, integrity, and ethical awareness (Den Hartog, 2015; Byun et al., 2018; Moore et al., 2019). In sum, by performing the roles of moral managers and moral people, ethical leaders strive to protect the rights and interests of various stakeholders of an organization, such as employees, organization, and society (Brown et al., 2005; Ahn et al., 2018; Zhang and Tu, 2018). The social learning theory offers a suitable perspective to explain the theoretical links between ethical leadership and OCBE. The social learning theory (Bandura, 1977, 1986) posits that an individual learns by observing and imitating his/her role models’ actions and behaviors.

Although research on OCBE is scarce, there is evidence of the positive influence of OCBE on organizations’ environmental performance that can help us address environmental issues, such as environmental degradation, global warming, and climate change (Magnus et al., 2012; Paillé et al., 2014; Boiral et al., 2015; Kennedy et al., 2015). Thus, given the importance of the OCBE for organizations’ environmental performance, the scarcity of research on how managers can encourage employees’ engagement in OCBE, and the growing emphasis to develop leadership models that encourage pro-environmental behaviors (Paillé et al., 2014; Afsar et al., 2016; Robertson and Barling, 2017; Luu, 2019; Tuan, 2019), we propose that ethical leadership is positively related to OCBE. The following arguments informed our proposition.

First, every economic activity always has a moral dimension (Follett, 1940). According to Barnett et al. (2005), businesses’ destructive influences on the natural environment and humankind can be countered through organizational members’ ethical behavior. Andrews (1980) argued that leaders’ ethical behaviors could play a pivotal role in addressing moral concerns linked with business organizations that deteriorate the quality of social life. As “the architect of purpose,” leaders can ensure that “the game is worth playing, the victory worth seeking, and life and career worth living” to ultimately protect the society from business activities’ detrimental effects (Andrews, 1980, p. 11). Ethical leaders act responsibly while interacting with employees, society, and the natural environment (Wu et al., 2015; Usman et al., 2018; Moore et al., 2019). Since ethics is the central tenant of ethical leadership, ethical leaders consider protecting the natural environment a moral obligation and demonstrate and promote pro-environmental behaviors (Wu et al., 2015), such as recycling and waste reduction to their followers. Drawing on social learning theory (Bandura, 1977, 1986), we propose that ethical leaders’ followers observe, learn from, and imitate their leaders’ sense of obligation toward the natural environment and humankind and engage in those discretionary behaviors that protect the natural environment.

Second, ethical leaders have a strong sense of social responsiveness, which is rooted in ethical leaders’ sense of responsibility to the society at large (Brown et al., 2005; Ahn et al., 2018; Bavik et al., 2018; Byun et al., 2018; Zhang and Tu, 2018). According to Zhu et al. (2014), ethical leaders assess the effect of their business decisions and operations on employees, organizations, customers, and the social and natural environment, as they aim to achieve a common good. Ethical leaders identify moral concerns linked with their business strategy and decisions and demonstrate pro-environmental behaviors through their business-related decisions, actions, and behaviors (Zhu et al., 2014). Thus, as environmental degradation poses a severe threat to the natural environment and humankind, we expect that ethical leaders will show responsiveness to such threats. Importantly, based on social learning theory (Bandura, 1977, 1986), the present study posits that followers learn and demonstrate social responsiveness through their actions and behaviors and engage in pro-environmental behaviors, such as energy conservation, recycling, and encouraging others to protect the natural environment.

Finally, OCBE is voluntary; it is linked with a sincere concern for the Planet that can only be demonstrated by individuals’ discretionary behaviors and actions aimed at improving humankind and nature (Paillé et al., 2014; Saeed et al., 2018; Testa et al., 2018; Tian and Robertson, 2019; Woosnam et al., 2019). According to Brown et al. (2005), concern for others (e.g., employees, the organization, consumers, and society) is one of the core features of ethical leadership. Ethical leadership’s feature of concern for others creates a moral commitment to engage in and promote a sense of ethical values (e.g., peace, ecology, and social justice) (Dolan et al., 2006) that positively shape pro-environmental behaviors (e.g., OCBE) (Dolan et al., 2006). The social learning theory (Bandura, 1977, 1986) proposes that ethical leaders’ demonstration of concern for others can instill moral commitment among followers to show concern for others (e.g., nature, and the society) that can positively influence employees’ engagement in OCBE. Thus, we predict a positive relationship between ethical leadership and OCBE.

It is important to note that ethical leadership in the present study refers to the respondents’ immediate supervisor. Our decision is based on the following key points. Although top management’s influence is the strongest on employees’ behaviors (Weaver et al., 2005; Mayer et al., 2009), the immediate supervisor has a unique relationship with his/her employees and usually maintains a close proximity and has frequent interactions with his/her subordinates (Davis and Rothstein, 2006; Johnson et al., 2010) that enhance the likelihood of supervisors’ influence on employees’ behaviors. Moreover, supervisors enact the top management’s policies, facilitate the penetration of the “tone at the top” throughout the organization, and often directly reward and discipline employees’ contribution or lack of it (Davis and Rothstein, 2006).

Hypothesis 1: Supervisors’ ethical leadership is positively related to OCBE.

### Green Psychological Climate as a Mediator

Green psychological climate entails employees’ shared perception that the organization’s environmental policies and procedures enhance environmental sustainability and green values (Ramus and Steger, 2000; Norton et al., 2014; Dumont et al., 2017; Zhou et al., 2018). Employees’ shared perception of the organizations’ policies, procedures, and practices are formed by the social cognitive processes (Bowen and Ostroff, 2004; Nishii et al., 2008; Kaya et al., 2010; Zientara and Zamojska, 2018). Social interactions enable employees to develop a shared perception of the organization’s practices and policies (Dumont et al., 2017; Norton et al., 2017; Zhou et al., 2018). In other words, employees’ interaction with their organization’s social environment and discussion about the organization’s practices and policies shape psychological climate.

Ethical leaders establish ethical standards and encourage their subordinates to follow these standards. By using two-way communication mechanisms, ethical leaders portray the importance of the established ethical standards to their subordinates and clarify employees’ ambiguities regarding the standards (Kalshoven and Den Hartog, 2009; Usman et al., 2018; Moore et al., 2019). Ethical leaders not only enforce the ethical standards through a punishment and reward system but also encourage their followers to raise their concerns regarding the ethical standards that promote an ethical culture in the organization (Kalshoven and Den Hartog, 2009; Zhang and Tu, 2018). As sustainability is an ethical issue (Barnett et al., 2005) and ethical leaders consider protecting the natural environment as a moral obligation (Wu et al., 2015), it is likely that ethical leaders develop and promote the environmental standards to protect the natural environment. The pro-environmental agenda of the organization not only promotes policies, procedures, and practices regarding environmental sustainability but also signals to employees that ethics and values are central to the organization (Rangarajan and Rahm, 2011).

Additionally, as ethical leaders encourage their followers to raise their concerns regarding the ethical standards (Kalshoven and Den Hartog, 2009; Ahn et al., 2018; Bavik et al., 2018), they are expected to persuade discussions among employees regarding the environmental standards (Kuenzi and Schminke, 2009). Such social interactions (the interaction of employees with their leaders, colleagues, and the context embedding these interactions) and discussions can shape employees’ shared perception that the organization’s environmental policies and procedures enhance environmental sustainability. Moreover, employees’ social interactions and discussions provide them with tacit guidelines about the nature and use of environmental standards when faced with a moral issue regarding the natural environment (Rangarajan and Rahm, 2011).

A green psychological climate not only promotes green behaviors but also inspires them to demonstrate discretionary, pro-social behaviors (Norton et al., 2017). Past research suggests that the psychological climate encourages employees to engage in pro-environmental behaviors (Kuenzi and Schminke, 2009; Dumont et al., 2017; Norton et al., 2017; Zhou et al., 2018; Zientara and Zamojska, 2018). As pro-environmental behaviors are voluntary in nature (Robertson and Barling, 2013, 2017), the present study predicts that green psychological climate enhances OCBE. Thus, we develop the following hypothesis.

Hypothesis 2: Green psychological climate mediates the positive relationship between supervisors’ ethical leadership and OCBE.

### Gender as a Moderator

Recent research on pro-environmental behaviors emphasizes that gender should be taken into account while analyzing employees’ behaviors toward the environment, as differences in beliefs, attitudes, and behaviors between men and women can have different manifestations of pro-environmental behaviors (Eisler et al., 2003; Xiao and McCright, 2015; Kennedy and Kmec, 2018; Vicente-Molina et al., 2018; Swim et al., 2019; Wang et al., 2019). Social role theory (House, 1981; Eagly, 1987; Eagly and Wood, 2012) suggests that gender-related differences in attitudes and behaviors are socially constructed and emerge due to the two related processes – social learning and societal power relations. Based on the social role theory, several scholars have suggested that gender-related differences in attitudes and behaviors are socially modeled through the reinforcement of societal power dynamics and status structures (Eagly and Steffen, 1984; Geis, 1993; Kidder, 2002). Likewise, gender role expectations shape and develop the pattern of individual behaviors, which are consistent with the cultural norms (Schahn and Holzer, 1990; Zelezny et al., 2000).

Conventional wisdom suggests that women are directed toward caregiver roles and are more passionate to learn and nurture pro-social behaviors, such as helping and showing concern for coworkers (Baumeister and Leary, 1995; Baumeister and Sommer, 1997; Kidder, 2002). Conversely, men are directed toward financial provider roles and have a strong economic orientation (Cross and Madson, 1997). Therefore, men often perform pro-social behaviors that are tied to formal rewards and status (Gabriel and Gardner, 1999; Gardner and Gabriel, 2004; Eagly and Wood, 2012). In this backdrop, drawing on the social role theory, we contend that, as compared to men, women are more likely to engage in discretionary behaviors that are not tied to the formal reward systems. Therefore, we speculate that women are more likely to engage in discretionary activities regarding nature than men. Indeed, a number of researchers have suggested that women demonstrate a stronger inclination to engage in pro-environmental, voluntary behaviors than men (Schahn and Holzer, 1990; Zelezny et al., 2000; Hechavarría, 2016; Vicente-Molina et al., 2018; Liu et al., 2019; Swim et al., 2019).

Additionally, as we have alluded before, ethical leaders can demonstrate pro-environmental behaviors, such as OCBE through their decisions, actions, and behaviors. According to the social learning theory (Bandura, 1977, 1986), ethical leaders’ pro-environmental behaviors would be imitated by their followers. Moreover, as we have argued above, women are more inclined to demonstrate pro-environmental behaviors than men, as is consistent with the social norms and role expectations. With this line of reasoning in mind, we understand that there is more congruence between the expected roles of an ethical leader and a woman. According to Markus (1977), an individual is more receptive and responsive to the information, actions, and behaviors that are congruent with his/her role and facilitate the performance of the role. Thus, it is likely that, as compared to men, women are more responsive to the ethical leaders’ pro-environmental standards and behaviors, suggesting that the effect of ethical leadership on green psychological climate and OCBE is different for men and women, where the effect for women may be stronger. Thus, we develop the following hypothesis.

Hypothesis 3: Gender moderates the indirect relationship (*via* green psychological climate) between supervisors’ ethical leadership and OCBE, such that the relationship is stronger for women.

## Materials and Methods

### Data Collection and Analysis

Data were collected from 447 employees in various manufacturing and service sector firms operating in China. The survey data were collected in three rounds, separated by a time lag of 2 months to avoid common method bias (Podsakoff et al., 2003). Initially, we contacted 1,000 alumni of a large public sector university in China through the alumni association of the university. The respondents were provided with a consent form and an information sheet. The aim and nature of our research, a promise of confidentiality, and the definitions of the key constructs were supplied in the information sheet. In the first round, data about ethical leadership and demographic variables (age, gender, education, and work experience – the number of years that a person has been employed) were collected. In the second and third rounds, data about green psychological climate and OCBE were collected, respectively. We received 513, 483, and 459 responses in the first, second, and third rounds, respectively. We discarded 11 responses that had missing data and, thus, we used 447 responses to test our hypotheses. To match the responses from the three rounds, a computer-generated code was placed on each questionnaire.

The respondents belonged to 440 different service and manufacturing companies spanning different industries, such as electronics, insurance, health, textile, leather, automobile manufacturing, telecommunication, chemical, engineering, and glass and ceramics. These alumni were working at different middle-level management positions in their respective organizations. The hierarchical breakdown of the respondents showed that 21, 29, and 50% were one level, two levels, and three levels below the top managers of their firms, respectively. The respondents were from diverse functional areas. A merchandizing manager, a manager of commercial knitting, and the head of the stitching department are examples of our respondents from the textile sector. An export manager, a quality control manager, and technical director are examples of the respondents from cement manufacturing firms. In terms of gender, the sample consisted of 279 (62.42%) males and 168 (37.58%) females. In terms of education, 195 (43.62%) employees had undergraduate degrees and 252 (56.38%) had completed master’s degrees. The average age and experience of the respondents were 36 years and 7.10 years, respectively.

Data were analyzed using SPSS 24.0 and AMOS 24.0. Descriptive statistics and correlations were performed using SPSS 24.0. Hypotheses of the present study were tested using structural equation modeling (SEM) and bootstrapping (AMOS 24.0).

### Measures and Variables

Unless otherwise stated, all the constructs were measured using a 5-point Likert scale anchored at 1 (strongly disagree) and 5 (strongly agree). Ethical leadership was measured using a 10-item scale (*α* = 0.88) from Brown et al. (2005). “My supervisor listens to what employees have to say” is a sample item. Green psychological climate was measured using a 5-item scale (*α* = 0.85) from Norton et al. (2014). “Our company believes it is important to protect the environment” was a sample item. OCBE was measured using a 13-item scale (*α* = 0.87) from Robertson and Barling (2017). “I help my co-workers be environmentally friendly at work” was a sample item.

## Results

### Means and Correlations

Means and correlations are presented in Table 1. All the correlations were in the expected direction. Both supervisors’ ethical leadership (*r* = 0.19, *p* < 0.01) and green psychological climate (*r* = 0.36, *p* < 0.01) correlated significantly with OCBE. Moreover, supervisors’ ethical leadership exhibited significant correlation with green psychological climate (*r* = 0.27, *p* < 0.01).

**Table 1:** Means and correlations.

Construct	Mean	SD	1	2	3
1. Supervisors’ ethical leadership	3.56	0.85			
2. Green psychological climate	3.57	0.86	0.27^**^		
3. OCBE	3.40	0.96	0.19^**^	0.36^**^	
4. Gender	1.38	0.48	−0.07	0.03	0.04

*n = 447; OCBE = organizational environmental citizenship behavior; SD = standard deviation*.

** *p < 0.01*.

### Measurement Model

Confirmatory factor analysis was used to evaluate the measurement model, which consisted of supervisors’ ethical leadership (SEL), green psychological climate (GPC), and organizational environmental citizenship behavior (OCBE). Four items, SEL9, SEL10, OCBE1, and OCBE7, were dropped, as they showed sub-optimal loadings. The fit indices (after dropping the four items), *χ*^2^ (249) = 645.53, *χ*^2^/df = 2.59, GFI = 0.90, IFI = 0.93, TLI = 0.92, CFI = 0.93, and RMSEA (IC-90%) = 0.05–0.06, show that the measurement model had a good fit with the data.

The values of average variance extracted (AVE), average shared variance (ASV), maximum shared variance (MSV), composite reliability (CR), and Cronbach’s alpha (*α*) are presented in Table 2. The scales showed satisfactory levels of internal consistency (*α* > 0.70) and reliability (CR > AVE > 0.50). The square root values of AVE for each variable in the study were greater than their inter-construct correlations. Moreover, ASV and MSV < AVE. Thus, the scales demonstrated satisfactory levels of internal consistency, discriminant validity, and convergent validity.

**Table 2:** Discriminant validity, convergent validity, and internal consistency.

Construct	1	2	3	α	CR	AVE	MSV	ASV
1. Supervisors’ ethical leadership	**0.71**			0.88	0.90	0.51	0.09	0.06
2. Green psychological climate	0.30	**0.74**		0.85	0.85	0.54	0.14	0.12
3. OCBE	0.20	0.38	**0.73**	0.87	0.92	0.53	0.14	0.09

*n = 447; OCBE = organizational environmental citizenship behavior; α = Cronbach’s alpha; CR = composite reliability; AVE = average variance extracted; MSV = maximum variance shared; ASV = average variance shared. Bold values on the diagonal of columns 2–4 are the square root values of AVE*.

### Structural Model

We evaluated structural models in three steps. In the first step, we looked at the direct association between supervisors’ ethical leadership and OCBE. We found a significant positive association between supervisors’ ethical leadership and OCBE (*β* = 0.21, *p* < 0.01). The *R*^2^ value for this model (1) was 0.04. The fit indices – *χ*^2^ (151) = 507.82, *χ*^2^/df = 3.36, GFI = 0.90, IFI = 0.92, TLI = 0.91, CFI = 0.92, and RMSEA (IC-90%) = 0.06–0.08 – showed that this initial structural model (model 1) had a satisfactory fit with the data. Thus, hypothesis 1 was supported.

**Figure 1:**
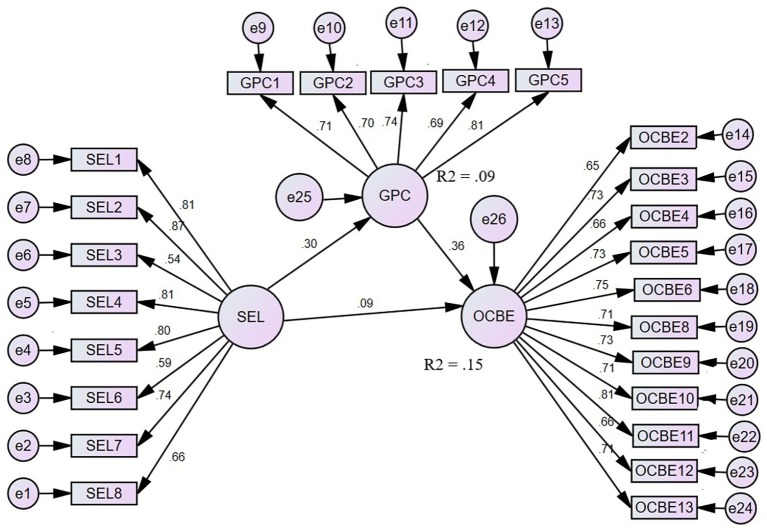
Structural model (2): Green psychological climate mediates the relationship between ethical leadership and OCBE.

In the second step (structural model 2 – mediation model, Figure 1), green psychological climate was introduced as the mediator of the relationship between supervisors’ ethical leadership and OCBE. The fit indices – *χ*^2^ (249) = 645.53, *χ*^2^/df = 2.59, GFI = 90, IFI = 0.93, TLI = 0.92, CFI = 0.93, and RMSEA (IC-90%) = 0.05–0.06 – show that the structural model (2) had a good fit with the data, suggesting that the green psychological climate as a mediator of the relationship between supervisors’ ethical leadership and OCBE was important.

Finally, we used bootstrapping by specifying a sample of size 2,000 in AMOS 24.0 to examine the significance of the role of the mediator. As already shown in the first step of our structural model, there was a significant direct relationship between supervisors’ ethical leadership and OCBE (*β* = 0.20, *p* < 0.01). The results of the mediated model (Table 3) show that after the inclusion of the mediator, the direct relationship between ethical leadership and OCBE became non-significant (*β* = 0.09, ns). Moreover, there was a significant indirect relationship between supervisors’ ethical leadership and OCBE *via* green psychological climate (*β* = 0.10, and 95% confidence interval did not overlap with zero, Table 3). Thus, hypothesis 2 was supported. That is, a green psychological climate mediated the positive relationship between supervisors’ ethical leadership and OCBE.

**Table 3:** Direct and indirect effects and 95% confidence intervals (model 2).

Parameter	Estimate	Lower	Upper
**Standardized direct effects**			
SEL → GPC	0.30^*^	0.18	0.42
SEL → OCBE	0.09	−0.01	0.19
GPC → OCBE	0.36^*^	0.23	0.47
**Standardized indirect effects**			
SEL → GPC → OCBE	0.10^*^	0.05	0.17

* *Empirical 95% confidence interval does not overlap with zero*.

*n = 447 (bootstrapping by specifying a sample of size 2,000); SEL = supervisors’ ethical leadership; GPC = green psychological climate; OCBE = organizational environmental citizenship behavior*.

### Moderated Mediation

The role of gender as the moderator of the relationship between supervisors’ ethical leadership and OCBE was tested using multigroup analysis, bootstrapping, and *χ*^2^ difference test. For bootstrapping, we specified a sample of size 2,000 at a 95% confidence interval. The gender variable was categorized into two groups – male (1) and female (2). The multigroup function in AMOS was used to estimate constrained and unconstrained models. The *χ*^2^ values and degrees of freedom of both the models were compared to examine the difference between the constrained and unconstrained models. The comparison showed a significant difference (*χ*^2^ difference = 42.06, df difference = 24) between the constrained and unconstrained models (Hair et al., 2010), suggesting that gender moderated that indirect relationship between supervisors’ ethical leadership and OCBE. The fit indices showed that the moderation model had a good fit with the data. The fit indices were *χ*^2^ (498) = 995.95, *χ*^2^/df = 2, IFI = 0.91, TLI = 0.90, CFI = 0.91, and RMSEA (IC-90%) = 0.04–0.05. The moderation results are presented in Table 4.

**Table 4:** Moderated mediation results.

Parameter	Estimate	Lower	Upper
Direct and indirect effects and 95% confidence intervals – Males			
Standardized direct effects			
SEL → GPC	0.23^*^	0.07	0.39
SEL → OCBE	0.11	−0.02	0.25
GPC → OCBE	0.28^*^	0.11	0.42
Standardized indirect effects			
SEL → GPC → OCBE	0.06^*^	0.02	0.16
Direct and indirect effects and 95% confidence intervals – Females			
Standardized direct effects			
SEL → GPC	0.42^*^	0.24	0.60
SEL → OCBE	0.03	−0.11	0.17
GPC → OCBE	0.51^*^	0.34	0.64
Standardized indirect effects	
SEL → GPC → OCBE	0.21^*^	0.12	0.33

* *Empirical 95% confidence interval does not overlap with zero. n = 447 (bootstrapping by specifying a sample of size 2,000)*.

*SEL = supervisors’ ethical leadership; GPC = green psychological climate; OCBE = organizational environmental citizenship behavior*.

The bootstrapping results of moderated models for both males and females (Table 4) show that the indirect relationship between supervisors’ ethical leadership and OCBE was significant for both males and females. Moreover, the direct relationship between supervisors’ ethical leadership and OCBE became non-significant for both males and females after introducing green psychological climate as the mediator. That is, the results indicate that green psychological climate significantly mediated the relationship between supervisors’ ethical leadership and OCBE for both males and females separately. However, the results of the heterogeneity test showed that the indirect relationship (*B* = 0.16, standard error = 0.05) between supervisors’ ethical leadership and OCBE for females was significantly stronger (*z* = 2.23, *p* < 0.05) than the indirect relationship (*B* = 0.04, standard error = 0.02) between supervisors’ ethical leadership and OCBE for males. Our hypothesis 3 predicted that gender moderates the indirect effect of supervisors’ ethical leadership on employees’ organizational citizenship behavior, where the effect for females is stronger. Thus, the results supported hypothesis 3.

## Discussion and Conclusion

### Theoretical Contributions

The work at hand makes several theoretical contributions. First, several scholars have suggested that environmental sustainability is non-obligatory in nature (Barnett et al., 2005; Testa et al., 2016; Tian and Robertson, 2019; Woosnam et al., 2019). According to Barnett et al. (2005), environmental sustainability is a moral value, and environmental issues are unethical in nature. Therefore, environmental sustainability can be achieved through leaders’ and employees’ ethical (Barnett et al., 2005) and pro-environmental behaviors, such as OCBE (Norton et al., 2017; Robertson and Carleton, 2018; Saeed et al., 2018; Testa et al., 2018; Zhou et al., 2018). Moreover, past research on ethical leadership shows that ethical leadership is positively related to employees’ ethical and pro-social behaviors and negatively related to employees’ unethical behaviors (Brown et al., 2005; Chughtai et al., 2015; Byun et al., 2018; Zhang and Tu, 2018; Moore et al., 2019), suggesting that ethical leadership can promote ethical and discretionary, pro-environmental behaviors.

However, empirical evidence about the relationship between ethical leadership and pro-environmental behaviors is scarce. Thus, by revealing a positive relationship between supervisors’ ethical leadership and OCBE, we extended the literature on ethical leadership and OCBE (Robertson and Barling, 2017). In line with the social learning theory (Bandura, 1977, 1986), we suggest that ethical leaders’ focus on ethics and their sense of responsibility toward society (Brown et al., 2005) and nature (Wu et al., 2015) can promote pro-environmental behaviors and reduce business organizations’ destructive effects on the natural environment. The findings indicate that due to their strong sense of social responsiveness toward society (Byun et al., 2018; Usman et al., 2018; Moore et al., 2019), ethical leaders identify and address moral concerns linked with their business strategy and operations. The findings suggest that ethical leadership’s features of concern for others (e.g., employees, the organization, consumers, and society) can create a moral commitment among employees to strive for ethical values (e.g., peace, ecology, and social justice) (Dolan et al., 2006) and demonstrate OCBE, such as recycling and conserving energy.

Second, we advanced the literature on green psychological climate (Robertson and Barling, 2013, 2017; Norton et al., 2017). Our study revealed the significant mediatory role of green psychological climate in the relationship between ethical leadership and OCBE. The findings suggest that ethical leaders consider protecting the natural environment as a moral obligation (Wu et al., 2015). Ethical leaders set ethical standards and use two-way communication mechanisms to portray the importance of the established ethical standards to their subordinates to shape employees’ shared perception that the organization’s policies and procedures are pro-environmental. Such a shared perception, in turn, inspires employees to engage in discretionary, pro-environmental behaviors. Thus, by establishing that green psychological climate mediates the positive relationship between ethical leadership and OCBE, our study contributed to and pulled together three important areas of knowledge: ethical leadership (Brown et al., 2005; Ahn et al., 2018; Bavik et al., 2018; Byun et al., 2018), green psychological climate (Norton et al., 2017; Zhou et al., 2018), and OCBE (Robertson and Barling, 2017; Saeed et al., 2018; Testa et al., 2018; Woosnam et al., 2019) and extended the nomological networks of antecedents and outcomes of OCBE and ethical leadership, respectively.

Finally, we contributed to the debate regarding the role of gender in pro-environmental behaviors (Eisler et al., 2003; Hechavarría, 2016; Vicente-Molina et al., 2018; Liu et al., 2019) by showing that gender significantly moderates the indirect relationship between ethical leadership and OCBE. We revealed that although green psychological climate significantly mediates the relationship between ethical leadership and OCBE for both males and females separately, the indirect relationship is stronger for females. Thus, our findings indicate that women engage more in discretionary activities toward nature than men. The finding concords with the literature (Zelezny et al., 2000; Hechavarría, 2016; Liu et al., 2019; Swim et al., 2019), which suggests that women demonstrate a stronger inclination to engage in pro-environmental, voluntary behaviors than men. Importantly, our study bought to the fore gender-based differences in the social learning process and the implications of these differences for OCBE. We suggest that, as there is more congruence between the ethical leaders’ and women’s expected roles, women are more responsive to their leaders’ pro-environmental behaviors than men.

### Practical Implications

By establishing the interrelations between ethical leadership, green psychological climate, and OCBE, our study offers several practical implications. First, supervisors’ ethical and pro-environmental behaviors can positively model employees’ pro-environmental behaviors and encourage them to engage in discretionary activities to deter the organization’s destructive effects on the natural environment. In this vein, we suggest that supervisors must understand their role as role models. Since employees tend to imitate and learn from their leaders’ behavior, supervisors should set examples of pro-environmental behaviors in an attempt to protect the natural environment.

We suggest that managers can also influence followers’ pro-environmental behaviors by establishing and implementing environmental standards. By establishing, promoting, and implementing the environmental standards, managers can create employees’ shared perception that organizational policies are pro-environmental. Such perceptions of green psychological climate are important antecedents of pro-environmental behaviors (recycling, conserving energy, and waste reduction) and can help managers overcome business-related threats to the natural environment. Importantly, managers need to appreciate women’s participation in the workforce, as women can be more effective in addressing environmental issues.

### Limitations and Future Research Directions

As with all research inquiries, the current research has a few limitations that should be noted. First, although the use of time-lagged data reduces the common method bias, it precludes any causal inferences. Future research should conduct longitudinal designs to establish casual relationships. Second, we collected data from China, the country in which environmental sustainability has gained much attention from the government policymakers that may have confounded the results. Future studies in other contexts can enhance our understanding of our hypothesized relationships. Third, although our hypothesized relationships were statistically significant, low *R*^2^ values indicate toward the complex nature of environmental sustainability, suggesting that there can be several factors that can affect, intervene with, and moderate the relationship between ethical leadership and OCBE. For instance, different leadership styles and green human resource practices (Saeed et al., 2018) can also shape employees’ OCBE. Likewise, harmoniously passionate employees have an enhanced sense of attention and assimilation (Ho et al., 2011) that increase the likelihood of imitating their leaders’ pro-environmental behaviors (Birkeland and Buch, 2015). The future researcher should consider employees’ harmonious passion as a potential enhancer of the relationship between ethical leadership and OCBE.

Finally, the additional mediators might be uncovered. The literature indicates that ethical leadership can shape employees’ moral attitudes (e.g., perceived accountability, moral efficacy, and moral intensity) (Arel et al., 2012; Lee et al., 2017) that, in turn, can lead to pro-environmental behaviors (Barnett et al., 2005). Thus, future research should investigate these variables as the potential mediators of the relationship between ethical leadership and OCBE.

## Data Availability

The datasets generated for this study are available on request to the corresponding author.

## Ethics Statement

The studies involving human participants were reviewed and approved by the ethics committee of the School of Management, Jiangsu University, China. The participants provided their written informed consent to participate in this study.

## Author Contributions

MK, DJ, MU, SS, and MA contributed to the definition of research objectives, models, and hypotheses, data analysis plan, and approval of the final manuscript. MA and MU contributed to the provision of materials (i.e., questionnaires). MK and DJ participated in data collection. MA, SS, and MU participated in data analysis and writing the main article. MK, DJ, and MA contributed to article revision and proofreading.

### Conflict of Interest Statement

The authors declare that the research was conducted in the absence of any commercial or financial relationships that could be construed as a potential conflict of interest.
